# Validity and reliability of the transculturally adapted Spanish version of the Implementation Leadership Scale (ILS)

**DOI:** 10.1186/s43058-023-00495-3

**Published:** 2023-09-12

**Authors:** Marta Llarena, Heather Lynn Rogers, Patricia Macia, Susana Pablo, Marta Gonzalez- Saenz de Tejada, Marta Montejo, Natalia Paniagua, Javier Benito, Mikel Rueda, Borja Santos, Gonzalo Grandes, Alvaro Sanchez

**Affiliations:** 1 Biocruces Bizkaia Health Research Institute, Network for Research on Chronicity, Primary Care, and Health Promotion (RICAPPS), Plaza Cruces s/n, 48903 Bizkaia, Barakaldo Spain; 2 Biocruces Bizkaia Health Research Institute, Plaza Cruces s/n, E-48903 Bizkaia, Barakaldo Spain; 3 Ikerbasque Basque Foundation for Science, Bilbao, Spain; 4 Biocruces Bizkaia Health Research Institute, Plaza Cruces s/n, 48903 Bizkaia, Barakaldo Spain; 5 Primary Care Research Unit of Bizkaia, Deputy Directorate of Healthcare Assistance, Biocruces Bizkaia Health Research Institute, Basque Healthcare Service – Osakidetza, Plaza Cruces s/n, 48903 Bizkaia, Barakaldo Spain; 6 Rontegi-Barakaldo Primary Care Center, University of the Basque Country, UPV/EHU, Biocruces Bizkaia Health Research Institute, Network for Research on Chronicity, Primary Care, and Health Promotion (RICAPPS), Bizkaia, Barakaldo Spain; 7 Pediatric Emergency Department, Cruces University Hospital, Biocruces Bizkaia Health Research Institute, Network for Research on Chronicity, Primary Care, and Health Promotion (RICAPPS), Plaza Cruces s/n, 48903 Bizkaia, Barakaldo Spain; 8 Pediatric Emergency Department, Cruces University Hospital, Biocruces Bizkaia Health Research Institute, University of the Basque Country, UPV/EHU, Network for Research on Chronicity, Primary Care, and Health Promotion (RICAPPS), Plaza Cruces s/n, 48903 Bizkaia, Barakaldo Spain; 9 Primary Care Research Unit of Bizkaia, Deputy Directorate of Healthcare Assistance, Biocruces Bizkaia Health Research Institute, Basque Healthcare Service – Osakidetza, Network for Research on Chronicity, Primary Care, and Health Promotion (RICAPPS), Plaza Cruces s/n, 48903 Bizkaia, Barakaldo Spain

**Keywords:** Implementation, Leadership, Validity, Reliability, Spanish ILS, Primary care

## Abstract

**Background:**

There is a need for pragmatic and reliable measures of sound factors that affect evidence-based practice (EBP) adoption and implementation in different languages and cultural environments. The Implementation Leadership Scale (ILS) is a brief and efficient measurement tool of strategic leadership for EBP implementation. The objective of this study was to assess the psychometric properties of the Spanish version of the ILS.

**Methods:**

The process of translation of the original ILS into Spanish consisted of forward translation, panel meeting, and back-translation. Scale face and content validity compared to that of the original version were assessed and ensured before agreement on the final version. Psychometric properties were examined in 144 healthcare professionals (family physicians, pediatricians, practice and pediatric nurses) involved in implementation or improvement research projects. ILS factor structure was tested by confirmatory factor analysis (CFA). Reliability was assessed by internal consistency analysis. The Pearson correlation between the ILS and the Organizational Support dimension of the Organizational Readiness for Knowledge Translation (OR4KT) questionnaire in the subsample of pediatricians and pediatric nurses (*n* = 52) was estimated for convergent validity analysis.

**Results:**

The CFA results indicated that the original four theorized first-order factors with a second-order Implementation Leadership factor fit the data well (χ^2^ = 107.70; *df* = 45;* p* < 0.001). All standardized first- and second-order factor loadings were statistically significant. Fit indexes showed acceptable figures (GFI = 0.90; CFI = 0.97; RMSEA = 0.10; SRMR = 0.053). Cronbach’s alpha coefficient for the four dimensions of ILS ranged from 0.90 to 0.97, while the reliability estimated for the total scale was 0.95. Results of convergent validity revealed high correlation (*r* = 0.56) between the ILS and the OR4KT’s Organizational Support dimension.

**Conclusion:**

The CFA results demonstrated that the tested first- and second-order factor structure of the 12-item Spanish version of the ILS is consistent with the factor structure of the original tool. The availability of the ILS will allow Spanish-speaking researchers to assess and advance understanding of the implementation leadership construct as a predictor of organizational implementation context.

**Supplementary Information:**

The online version contains supplementary material available at 10.1186/s43058-023-00495-3.

Contributions to the literature

- There is a need for pragmatic and reliable measures in different languages and cultural environments regarding factors that affect EBP adoption and implementation, such as the construct of leadership.
- Our study is the first to transculturally adapt the ILS to Spanish as well as demonstrate its psychometric properties with a sample of both physicians and nurses.
- We provide a Spanish version of ILS for the Spanish-speaking scientific community, which will allow researchers to assess and advance understanding of the implementation leadership construct as a predictor of organizational implementation context.

## Background

Implementation research is defined as the scientific study of methods to promote the systematic uptake of research findings and other evidence-based practices (EBPs) into routine practice, and, hence, to improve the quality and effectiveness of health services [1]. It includes the study of influences on healthcare professionals and organizational behavior [1]. Factors influencing EBP implementation success include individual provider factors as well as organizational factors (i.e., organizational culture, climate, and leadership) that are expected to have an even more profound effect on EBP integration [2]. Of these factors, leadership has been identified as one of the most significant components of the organizational context that may act as a mechanism for improving EBP implementation [3, 4] and therefore impact on organizational change and innovation within healthcare organizations [5].

Leadership at several organizational levels, from top to first-level leaders, facilitates relevant mechanisms in promoting implementation, such as positive organizational climate in the workplace [6], positive employee attitudes towards work [7] and adopting EBPs [8], positive trusting inter-professional relationships and supportive collaborative working vision [9], and commitment to organizational change [10]. Despite the critical role of first-level leaders, those who directly supervise and manage direct service providers, in the translation of clinical research and practice change, little is known about how to identify and measure leadership behaviors in health professionals [11]. In this regard, Aarons et al. developed the Implementation Leadership Scale (ILS) in order to assess the degree to which leaders engage in specific leadership behaviors that are critical for effective EBP implementation [12]. The scale has four dimensions: (1) proactive leadership, which addresses the degree to which a leader establishes clear goals and plans as well as removes obstacles to EBP implementation; (2) knowledgeable leadership, which measures the degree to which a leader has a deep understanding of EBP and is able to answer staff’s EBP implementation-related questions; (3) supportive leadership, which represents the degree of the leader’s support and recognition of staff’s efforts to learn, use and adopt EBPs; and (4) perseverant leadership, which reflects the degree to which a leader is consistent, unwavering and responsive to the ups and downs, challenges and issues that arise in the EBP implementation process [12].

The ILS was originally developed and tested in mental health settings [12] and has then been cross-validated and used in substance use facilities [13], child welfare service organizations [14], education sectors [15], and acute care in nursing contexts [16], as well as with mental health clinic supervisors [17]. Nonetheless, to the best of our knowledge, so far, the ILS has been only translated into Chinese [18, 19], Greek [20], Norwegian [21], and German [22]. Hence, we consider that there is a need for pragmatic and reliable measures of sound factors that affect EBP adoption and implementation in different cultural environments and languages, including Spanish.

In this context, the main objective of this study was to assess the psychometric properties of a Spanish version of the Implementation Leadership Scale, in order to evaluate whether this version measures in the same way and with the same assurance as the original English version.

## Methods

### Participants and procedure

Participants were family physicians, pediatricians, practice nurses, and pediatric nurses, who were involved in implementation or improvement research projects conducted across the 13 Integrated Healthcare Organizations (IHOs) of Osakidetza – Basque Health Service (References: PI15/00350 and PI19/00234). These projects involved an implementation strategy that included leadership promotion aimed at fostering both the knowledge and skills regarding the EBP of interest as well as the capacity to lead the EBP implementation process in order to promote the EBP’s translation and integration into routine practice, of one of the practice healthcare professionals who was elected as leader by consensus within the collaborating centers. The present sample responds to a convenience sampling method as it consisted of professionals who responded to several procedures via online surveys used to evaluate the implementation or improvement projects in which they were involved. All these online surveys provided information about the main objectives of the study, data confidentiality, and the planned use of the resulting data. All participants voluntarily provided their written informed consent before taking part in the study. The studies within which these surveys were planned and conducted were reviewed and approved by the Basque Country Clinical Research Ethics Committee (CEIm Euskadi) (References: 08/2015 and 07/2019).

Osakidetza—Basque Health Service provides universal coverage and services are free at the point of use, aside from co-payment for drugs, funded through regional general taxation. Primary, specialized, and social health-related service provision is organized around 13 IHOs, with 135 primary care health centers and 9 hospitals, that cover the 3 provinces of the region of the Basque Country: Araba, Bizkaia, and Gipuzkoa. Each resident is on the list of one family physicians or pediatrician who offers comprehensive primary care and refers patients for hospital and specialty services. Services users can also attend emergency care by a point of care service provided by hospitals.

### Instruments

#### Implementation leadership scale (ILS)

The ILS consists of four factors: 1) Proactive leadership, 2) Knowledgeable leadership, 3) Supportive leadership, and 4) Perseverant leadership [12]. Each factor is assessed with three items, which are scored on a 0 (“not at all”) to 4 (“to a very great extent”) scale. The score obtained indicates the degree to which a leader/supervisor is engaged in the abovementioned behaviors with regard to EBP implementation. There are two versions of the ILS, one for staff to report about leader/supervisor, and another for leaders/supervisors to report about themselves. The staff version of the ILS was used in this study. The score for each subscale is created by computing a mean score for each set of items on a given subscale. The mean score for the total ILS is obtained from the mean of the four subscale scores.

#### Organizational readiness for knowledge translation (OR4KT) questionnaire

The Organizational Support dimension of the Spanish version of the OR4KT questionnaire was used for the assessment of convergent validity [23, 24]. This questionnaire was developed as a tool for assessing healthcare organizational readiness to change clinical practice in order to enable EBP implementation and adoption of proven interventions. The OR4KT questionnaire was originally developed in English and then translated into Spanish and French [24]. The questionnaire consists of 59 items that assess 6 dimensions and 23 sub-dimensions related to organizational predisposition to knowledge translation: organizational climate, organizational support, contextual factors, change content, leadership, and motivation. Organizational support includes four sub-dimensions: support climate (items 40 to 43), monitoring (items 44 and 45), evaluation process (items 46 to 48), and feedback (item 49). Each item is scored on a five-point Likert scale from 1 (“strongly disagree”) to 5 (“strongly agree”). The total score is computed by summing the scores on each item, with a maximum score of 295 points. Then, this score is normalized on a 0 to 100 scale to ease interpretation.

### Transcultural adaptation procedure, and face and content validity assessment

The ILS original version was translated into Spanish by a translation/back-translation procedure [25]. The process consisted of the following steps: forward translation, panel meeting, back-translation, assessment of face and content validity and agreement on a final version.

In the first step, the original ILS was translated into Spanish by experienced native Spanish-speaker translators who were members of the research team (AS, PM). Special attention was paid to the readability and clarity of the items. In the second step, the translated Spanish version was reviewed by bilingual members of the research team including the translators (GG, SP, AS, PM) seeking to detect any inadequate expressions/concepts of the translation and discrepancies between the forward translation and the existing English version. In the third step, an independent native English speaker (HR), who was fluent in Spanish and had no knowledge of the original ILS, performed the back-translation. The original English version and the back-translation were compared and the conceptual and cultural equivalence of the transculturally-adapted version was assessed, placing special emphasis on the linguistic equivalence. There were only small discrepancies which were discussed until consensus was reached concerning minor modifications required.

Finally, a group of researchers from the Primary Care Research Unit of Bizkaia, who were not involved in the present study or in the implementation or improvement projects within which we sought to validate the Spanish ILS, evaluated the extent to which the translated version maintained the face and content validity of the original version and assessed the clarity of the wording of the items. Minor final corrections were made based on researchers’ comments. The final Spanish version of the ILS instrument used in the validation process is presented in Additional file 1.

### Statistical analysis

Confirmatory factor analysis (CFA) was conducted to confirm the ILS factor structure. In a first step, we fitted a model in which we specified which sub-dimension each of the items belonged to. In a second step, we carried out a second-order analysis, checking the fit of a model in which each sub-dimension was loaded onto its reference dimension and these, in turn, onto a global dimension that measures implementation leadership. The model fit was assessed using the value of the chi-square/degree of freedom ratio (χ^2^/*df*) and also the comparative fit index (CFI), the goodness of fit index (GFI), the root mean square error of approximation (RMSEA), and the standardized root mean square residual (SRMR). Values of χ^2^/*df* ≤ 2.5, CFI ≥ 0.95, GFI ≥ 0.90, RMSEA ≤ 0.06, and SRMR ≤ 0.08 indicate acceptable model fit [26]. Reliability was quantified in terms of consistency and Cronbach’s alpha coefficients were calculated to estimate internal consistency for each of the subscales and the total scale. A value of α ≥ 0.7 was considered good [27]. Pearson coefficients were used to assess convergent validity of the ILS total scale and four subscales with the Organizational Support dimension, and its four sub-dimensions, of the OR4KT questionnaire. The correlation is considered high when this coefficient is greater than 0.5, and moderate when the coefficient is > 0.3 [28]. SAS (v. 9.2, SAS Institute, Cary, NC, USA) and IBM SPSS (version 23.0; Armonk, NY, USA) were used to perform all the statistical analysis.

## Results

Translation of the ILS tool into Spanish and back-translation into English did not encounter any major content or language issues. Each of the items were precisely and comprehensibly worded and completely captured the concept addressed by the original one, resulting in a Spanish version of the ILS conceptually equivalent to the English instrument. The psychometric properties of the Spanish ILS were examined in a sample of 144 healthcare professionals that consisted of family physicians (*n* = 51; 35.4%), pediatricians (*n* = 38; 26.4%), practice nurses (*n* = 48; 33.3%) and pediatric nurses (*n* = 7; 4.9%). Out of them, 117 professionals (81.3%) were from a total of 33 primary care health centers and 27 (18.7%) from 5 specialized care settings across 12 out of the 13 IHOs of Osakidetza – Basque Health Service. The mean age of participants was 48.34 years (SD = 9.66; range 25–65) and the majority were female (81.3%).

The descriptive and reliability statistics for the total scale and the four subscales of the ILS are reported in Table 1. The total scale mean was 3.34 (SD = 0.58), whereas means for the four subscales were between 3.20 and 3.44. Cronbach’s alpha coefficients showed very good internal consistency for the four dimensions of the ILS, with values ranging from 0.90 to 0.97, while the reliability estimated for the total scale was 0.95.

**Table 1:** Descriptive and reliability statistics of the Spanish version of the Implementation Leadership Scale (ILS)

ILS items, subscales and total	Mean	SD	α
**1. Proactive leadership**	**3.20**	**0.69**	**0.90**
Removed obstacles to implementation of EBP	3.26	0.77	
Established clear standards for implementation EBP	3.10	0.77	
Developed a plan to facilitate EBP implementation	3.24	0.74	
**2. Knowledgeable leadership**	**3.43**	**0.64**	**0.92**
Is knowledgeable about EBP	3.35	0.76	
Is able to answer staff questions about EBP	3.42	0.67	
Knows what he/she is talking about when it comes to EBP	3.52	0.60	
**3. Supportive leadership**	**3.44**	**0.68**	**0.97**
Supports employee efforts to learn more about EBP	3.44	0.67	
Recognizes and appreciates employee efforts	3.44	0.73	
Supports employee efforts to use EBP	3.44	0.70	
**4. Perseverant leadership**	**3.30**	**0.71**	**0.94**
Perseveres through the ups and downs of implementing	3.33	0.75	
Carries on through the challenges of implementing EBP	3.31	0.73	
Reacts to critical issues regarding implementation of EBP	3.25	0.79	
**ILS total**	**3.34**	**0.58**	**0.95**

*n* = 144; *ILS* Implementation leadership scale, *EBP* Evidence-based practice, *M* Mean, *SD* Standard deviation; α, Cronbrach’s alpha

**Fig. 1:**
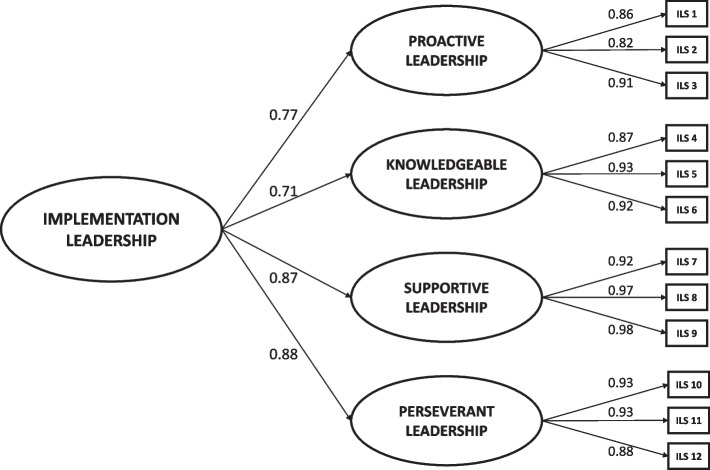
Second-order confirmatory factor analysis factor loadings for the Spanish version of the Implementation Leadership Scale (ILS). Note: *n* = 144; All factor loadings are standardized and are statistically significant, *p* < 0.001; χ.^2^ = 107.70, *df* = 45,* p* < 0.001; GFI = 0.90; CFI = 0.97; RMSEA = 0.10; SRMR = 0.053

The CFA confirmed the already designed factor structure of the ILS. In this regard, the CFA results indicated that the original four theorized first-order factors with a second-order Implementation Leadership factor fit the data well (χ^2^ = 107.70; *df* = 45; *p* < 0.001). Moreover, fit indexes showed acceptable figures (GFI = 0.90; CFI = 0.97; RMSEA = 0.10; SRMR = 0.053). Figure 1 displays the standardized factor loadings for the higher-order factor model. First-order factor loadings ranged from 0.82 to 0.98, while second-order factor loadings ranged from 0.71 to 0.88. All standardized first- and second-order factor loadings were statistically significant (*p* < 0.001).

Convergent validity was assessed in the subsample of pediatricians and pediatric nurses (*n* = 52). As expected, convergent validity analysis revealed high correlation (Pearson coefficient, *r* = 0.56) between the ILS total scale and the Organizational Support dimension of the OR4KT questionnaire. In addition, the analysis between the OR4KT’s Organizational Support dimension and two of the ILS subscales (proactive and perseverant leadership) showed high correlation (*r* = 0.51 and *r* = 0.54, respectively). Moreover, ILS total scale also displayed high correlations (*r* = 0.52) with the sub-dimensions support climate and evaluation process of the OR4KT’s Organizational Support dimension (Table 2).

**Table 2:** Pearson correlations of the Spanish Implementation Leadership Scale (ILS) scores with the Organizational Support dimension of the Organizational Readiness for Knowledge Translation (OR4KT) questionnaire scores

	**Implementation leadership scales**				
	**Proactive**	**Knowledgeable**	**Supportive**	**Perseverant**	**ILS total**
**OR4KT**					
**Organizational support**	0.51^**^	0.45^**^	0.40^**^	0.54^**^	0.56^**^
Support climate	0.48^**^	0.43^**^	0.37^**^	0.48^**^	0.52^**^
Monitoring	0.39^**^	0.32^*^	0.25	0.41^**^	0.41^**^
Evaluation process	0.46^**^	0.41^**^	0.39^**^	0.49^**^	0.52^**^
Feedback	0.37^**^	0.32^*^	0.33^*^	0.46^**^	0.44^**^

*n* = 52; *ILS* Implementation leadership scale, *OR4KT* Organizational Readiness for Knowledge Translation questionnaire, *p < 0.05, **p < 0.01

## Discussion

This study aimed to assess the psychometric properties of the Spanish version of the Implementation Leadership Scale (ILS). The ILS is a brief and efficient measurement tool of strategic leadership for EBP implementation. The instrument consists of 12 items grouped under four dimensions assessing the degree to which a leader is proactive, knowledgeable, supportive, and perseverant in the implementation of EBP. Firstly, we developed a linguistically equivalent version in Spanish of the original English version of the ILS that was developed by Aarons et al. [12]. After having established the face and content validity of the Spanish version, we proceeded with the validation process by assessing its factor structure and psychometric properties (reliability and validity) in the context of implementation or improvement projects conducted in Osakidetza – Basque Health Service.

The results of the initial validation process indicated that the Spanish ILS is a reliable instrument based on the internal consistency assessment. The Cronbach’s alpha coefficient for the total scale is 0.95, which provides strong support for the reliability of this tool. This value is comparable to that obtained in the assessment of the original English version of the ILS (α = 0.98) [12] and in other cross-validation studies using the employee ratings (α = 0.97; 0.99 and 0.98) [13, 15, 16] or the leader self-ratings (α = 0.95) [22]. When compared to the other available translations of the ILS (i.e., the Chinese [18, 19], Greek [20], Norwegian [21], and German [22] versions), the internal consistency of the Spanish ILS is as excellent as the Norwegian ILS, with Cronbach’s alpha coefficients between 0.93 and 0.97 for the four dimensions and 0.96 for the total scale [21]. In contrast, the Chinese, Greek, and German versions of ILS showed good/adequate reliability with Cronbach’s alpha coefficients of 0.86–0.95 for the four subscales and 0.93 for total scale of the Chinese ILS [19]; 0.85–0.91 for the four subscales and 0.94 for total scale of the Greek ILS [20], and 0.875 for the total scale and of 0.77–0.92 for the four factors of the German ILS [22].

Secondly, to assess the validity of the Spanish version of the ILS, we performed CFA to provide evidence of internal structure validity. The CFA results confirmed a good model fit to the theorized first- and second-order factor structure of the Spanish ILS as previously reported in several studies in different settings, as well as showing acceptable factor loadings [12–17, 19–21]. Our findings demonstrated the factor structure of the Spanish version of the ILS as well as its structural validity in the context of primary care and pediatrics departments (both primary and specialized care) when completed by family physicians, pediatricians, practice nurses, and pediatric nurses.

Most ILS validation studies have used the Multifactor Leadership Questionnaire (MLQ) [29] to evaluate convergent validity, reporting that the ILS had moderate to high correlations with the MLQ total and subscales [12, 13, 19, 21]. However, other scales have been used for assessing convergent validity, as for example the Quality of leadership dimension of the Copenhagen Psychosocial Questionnaire Version II (COPSOQ II) [30] in the Greek ILS validation study (*r* = 0.792) [20]. In our study, we have used the Organizational Support dimension of the OR4KT questionnaire, which measures an organization’s level of readiness to implement EBPs; and is available in Spanish, having been developed, translated into Spanish, and transculturally validated by members of our research group [23, 24]. Specifically, we have focused on the Organizational Support dimension which assesses whether there is a supportive climate within a healthcare center, and the reception of monitoring, evaluation processes, and feedback, all of them reflecting the behaviors expected to be performed by the leader as part of the implementation strategy in the projects involved in the present validation study. In short, we have observed a high correlation (*r* = 0.56) between the Spanish ILS total scale and the Organizational Support dimension of the OR4KT questionnaire, indicating good convergent validity in our clinical setting; though this was only assessed in the subsample of pediatricians and pediatric nurses (*n* = 52).

Our study contributes to the literature not only by demonstrating for the first time the psychometric properties of the ILS in Spanish but also by including for the first time results from a Spanish sample of both physicians and nurses in a primary care setting (81.3% out of the total sample reported working in primary care health centers). To our knowledge, the ILS has not been validated with physicians yet [11]; and therefore our study is the first to have included physicians (61.8% out of the total sample) who rated their leaders. Our findings are in line with the results from previous studies in other languages and in different settings where the staff version of ILS was validated among clinicians working at mental health clinics in the USA [12] and Norway [21]; service providers at substance use disorder treatment agencies [13]; employees at child welfare service organizations [14]; employees in the education sector [15]; nurses and midwives in Greece [20] and nurses in the USA and China [16, 19]. Nonetheless, it should be pointed out that in this study, we validated the Spanish staff version of the ILS. Since discrepancies were previously reported between supervisor and clinician/follower ratings [31–33], our results would not be comparable with the assessment of the German ILS, where they validated the leader version with physicians [22], or with a previously reported cross-validation study of the ILS with mental health clinic supervisors’ self-ratings [17].

A limitation of the current study was the final sample size (*n* = 144), which was somewhat smaller than that in previous validation studies, which included above 200 participants [12–14, 16, 19, 21]. However, it should be noted that similar good results with regard to the factor structure and the internal consistency (reliability) were obtained in our study. Second, the data were collected from a sample consisting of family physicians, pediatricians, practice nurses, and pediatric nurses and not from other specialties. On the other hand, the fact that the study was conducted in 12 out of the 13 IHOs of Osakidetza – Basque Health Service and that the participants were from numerous different primary care health centers could contribute to its generalizability.

Future research should be considered in order to evaluate the properties of the Spanish version of the ILS among different healthcare professionals as well as in different regions in Spain and Spanish-speaking countries with different cultural environments. Our aim is to promote implementation research in the Spanish-speaking scientific community by the development of implementation research tools to support the design, evaluation and reporting of implementation research projects in non-English speaking context.

## Conclusions

There is a strong need for pragmatic, efficient measures that assess the construct of leadership in the context of EBP implementation in different languages and cultural environments. This study led to the translation, transcultural adaptation, and assessment of face and content validity, internal structure validity, and psychometric properties of the Spanish version of the ILS. The CFA results demonstrated that the tested first- and second-order factor structure of the 12-item Spanish version of the ILS is consistent with the factor structure of the original tool. The availability of the Spanish version of this instrument will allow Spanish-speaking researchers to assess and advance understanding of the implementation leadership construct as a predictor of organizational implementation context.

### Supplementary Information

**Additional file 1. **Spanish version of the Implementation Leadership Scale (ILS) – Staff version (PDF format).

## Data Availability

The datasets generated and analyzed during the current study are available from the corresponding author on reasonable request.

## Abbreviations

CFA: Confirmatory factor analysis
CFI: Comparative Fit Index
EBP: Evidence-based practice
GFI: Goodness of Fit Index
IHO: Integrated healthcare organization
ILS: Implementation Leadership Scale
M: Mean
OR4KT: Organizational Readiness for Knowledge Translation
RMSEA: Root mean square error of approximation
SD: Standard deviation
SRMR: Standardized root mean square residual
χ^2^/*df*: Chi-square/degree of freedom ratio
