# Supplementary material for: Validity and reliability of the transculturally adapted Spanish version of the Implementation Leadership Scale (ILS)
Source: Implement Sci Commun. 2023 Sep 12;4:112. doi: 10.1186/s43058-023-00495-3 (PMC10496227; doi:10.1186/s43058-023-00495-3)
Supplement: Supplementary file 1 — Additional file 1. Spanish version of the Implementation Leadership Scale (ILS) – Staff version (PDF format). [file 43058_2023_495_MOESM1_ESM.pdf]

ESCALA DE LIDERAZGO EN IMPLEMENTACION (ELI) - Versión para el personal

La Escala de Liderazgo en Implementación valora el grado en el que un/a líder es proactivo/a, conocedor/a, da apoyo y es constante con respecto a la implementación de la práctica basada en la evidencia.

Por favor, indique el grado en el que está de acuerdo con cada una de las siguientes afirmaciones.

0	1	2	3	4
**En absoluto**	**Ligeramente**	**Moderadamente**	**Bastante**	**Mucho**

**Proactivo/a**	**0**	**1**	**2**	**3**	**4**
1. [*Nombre del supervisor/a*] ha desarrollado un plan para facilitar la implementación de la práctica basada en la evidencia	☐	☐	☐	☐	☐
2. [*Nombre del supervisor/a*] ha eliminado obstáculos para la implementación de la práctica basada en la evidencia	☐	☐	☐	☐	☐
3. [*Nombre del supervisor/a*] ha establecido estándares claros en el servicio para la implementación de la práctica basada en la evidencia	☐	☐	☐	☐	☐

**Conocedor/a**	**0**	**1**	**2**	**3**	**4**
4. [*Nombre del supervisor/a*] es un/a entendido/a en la práctica basada en la evidencia	☐	☐	☐	☐	☐
5. [*Nombre del supervisor/a*] es capaz de responder a las preguntas sobre la práctica basada en la evidencia	☐	☐	☐	☐	☐
6. [*Nombre del supervisor/a*] sabe de lo que habla cuando se refiere a la práctica basada en la evidencia	☐	☐	☐	☐	☐

**Apoyo**	**0**	**1**	**2**	**3**	**4**
7. [*Nombre del supervisor/a*] reconoce y aprecia los esfuerzos de los/las trabajadores/as para conseguir una implementación exitosa de la práctica basada en la evidencia	☐	☐	☐	☐	☐
8. [*Nombre del supervisor/a*] apoya los esfuerzos de los/las trabajadores/as para aprender más sobre práctica basada en la evidencia	☐	☐	☐	☐	☐
9. [*Nombre del supervisor/a*] apoya los esfuerzos de los/las trabajadores/as para usar práctica basada en la evidencia	☐	☐	☐	☐	☐

**Constante**	**0**	**1**	**2**	**3**	**4**
10. [*Nombre del supervisor/a*] persevera frente a los altibajos que conlleva la implementación de la práctica basada en la evidencia	☐	☐	☐	☐	☐
11. [*Nombre del supervisor/a*] continua adelante frente a los retos de la implementación de la práctica basada en la evidencia	☐	☐	☐	☐	☐
12. [*Nombre del supervisor/a*] reacciona a los aspectos críticos de la implementación de la práctica basada en la evidencia abordando los problemas de una manera abierta y eficaz	☐	☐	☐	☐	☐
